# Supplementary choline attenuates olive oil lipid emulsion‐induced enterocyte apoptosis through suppression of CELF1/AIF pathway

**DOI:** 10.1111/jcmm.13430

**Published:** 2017-11-06

**Authors:** Jun‐Kai Yan, Jie Zhu, Zi‐Zhen Gong, Jie Wen, Yong‐Tao Xiao, Tian Zhang, Wei Cai

**Affiliations:** ^1^ Xin Hua Hospital affiliated to Shanghai Jiao Tong University School of Medicine Shanghai China; ^2^ Shanghai Key Laboratory of Pediatric Gastroenterology and Nutrition Shanghai Institute for Pediatric Research Shanghai China

**Keywords:** total parenteral nutrition, intestinal atrophy, olive oil lipid emulsion, choline, apoptosis, Caco‐2, CELF1, AIF

## Abstract

Enterocyte apoptosis induced by lipid emulsions is a key cause of intestinal atrophy under total parenteral nutrition (TPN) support, and our previous work demonstrated that olive oil lipid emulsion (OOLE) could induce enterocyte apoptosis *via* CUGBP, Elav‐like family member 1 (CELF1)/ apoptosis‐inducing factor (AIF) pathway. As TPN‐associated complications are partially related to choline deficiency, we aimed to address whether choline supplementation could attenuate OOLE‐induced enterocyte apoptosis. Herein we present evidence that supplementary choline exhibits protective effect against OOLE‐induced enterocyte apoptosis both *in vivo* and *in vitro*. In a rat model of TPN, substantial reduction in apoptotic rate along with decreased expression of CELF1 was observed when supplementary choline was added to OOLE. In cultured Caco‐2 cells, supplementary choline attenuated OOLE‐induced apoptosis and mitochondria dysfunction by suppressing CELF1/AIF pathway. Compared to OOLE alone, the expression of CELF1 and AIF was significantly decreased by supplementary choline, whereas the expression of Bcl‐2 was evidently increased. No obvious alterations were observed in Bax expression and caspase‐3 activation. Mechanistically, supplementary choline repressed the expression of CELF1 by increasing the recruitment of CELF1 mRNA to processing bodies, thus resulting in suppression of its protein translation. Taken together, our data suggest that supplementary choline exhibits effective protection against OOLE‐induced enterocyte apoptosis, and thus, it has the potential to be used for the prevention and treatment of TPN‐induced intestinal atrophy.

## Introduction

Intestinal atrophy is one of the common complications during total parenteral nutrition (TPN) which provides all nutrition intravenously for preterm infants, patients with intestinal failure and those who cannot tolerate enteral nutrition (EN) 1, 2, 3. However, the pathogenesis of TPN‐induced intestinal atrophy has not been fully elucidated, and thus, it still lacks effective therapies and appropriate preventative treatment in clinical practice currently, leading to delay in the achievement of enteral autonomy. Recently, a rodent study has shown that enterocyte apoptosis induced by intravenous lipid emulsions (LE) may be one of the key causes of intestinal atrophy under TPN support, suggesting that the significance of LEs is no longer limited to ‘energy supply’, beyond which it may also function as a key regulator of intestinal homeostasis 4.

Currently, the commercially available LEs for clinical use with various composition of fatty acids include: fish oil‐derived lipid emulsion (FOLE), soya bean oil‐derived lipid emulsion (SOLE) and 80% olive oil‐supplemented lipid emulsion (OOLE), which predominantly contains *n*‐3, *n*‐6 and *n*‐9 unsaturated fatty acids, respectively. Previously, we found a new caspase‐independent pathway (CELF1/AIF pathway) that specifically mediates OOLE‐induced apoptosis in Caco‐2 cells 5. CUGBP, Elav‐like family member 1 (CELF1) is one of the RNA binding proteins that contribute to the control of cell apoptosis and proliferation, as demonstrated by a number of recent studies both *in vivo* and *in vitro* 6, 7, 8, 9. We found that OOLE increases the expression of CELF1, and in turn induces apoptosis through apoptosis‐inducing factor (AIF). In that study, we provided a new perspective on the molecular mechanisms underlying OOLE‐induced enterocyte apoptosis, but it also suggested that preventative treatment targeting CELF1 needs to be further studied. Given that the application of LEs is extremely restricted in clinical use currently; hence, the key question turns to be whether it is possible to minimize the side effect of LEs by optimizing their composition with small modifications.

As known, choline is a water‐soluble, lipotropic, quaternary amine that is an essential structural component of cell membranes, the neurotransmitter acetylcholine and phospholipid biosynthesis, and it participates in various biological functions, including the regulation of cell proliferation, death and transformation 10; lipid metabolism 11; signalling transduction 12; mitochondrial bioenergetics 13; and epigenetic regulation of DNA, RNA and protein as a methyl donor 14, 15. The American Society for Parenteral and Enteral Nutrition recommends the development of choline products for parenteral nutrition 16, as evidence is accumulating that plasma‐free choline concentration is below normal in patients receiving long‐term TPN, contributing to the pathogenesis of TPN‐associated complications 17, 18. Taken together with the fact that choline deficiency activates cellular apoptosis 19, 20, 21 partially owing to defective cellular repair mechanisms, these findings make it an attractive point to address whether supplementation of choline is beneficial for alleviating OOLE‐induced enterocyte apoptosis under TPN support. To address this, we used a rat model of TPN and hypothesized that attenuation in the enterocyte apoptosis would be seen *in vivo* when supplementary choline was added to existing OOLE. Moreover, we also used Caco‐2 cells as a model which has been approved suitable for studying TPN ingredients *in vitro* previously elsewhere 22, 23 and investigated the changes in the expression of CELF1 and AIF by supplementary choline. Our results may be helpful to develop new preventative treatment against OOLE‐induced enterocyte apoptosis under TPN support.

## Material and methods

### TPN rat model

A total of 30 male Sprague‐Dawley specific‐pathogen‐free rats (3 weeks old; 70 ± 5 g, obtained from the Experimental Animal Center of Shanghai Jiaotong University School of Medicine) were maintained in an environment controlled at room temperature (25°C ± 0.5°C) and a relative humidity (40–60%) in a 12‐hrs light and 12‐hrs dark cycle. Rats were initially fed *ad libitum* with standard chow and water and allowed to acclimate for 1 week prior to surgery. Cannulation for TPN was identical to that previously described 24. The rats were then randomly assigned to three groups: TPN, TPN with supplementary choline (TPN +choline) and a saline control group (sham) for 7 days. The TPN group received TPN delivery (400 kcal/kg/d) continuously for 7 days but was allowed water *ad libitum*. The TPN+choline group received the same TPN solution with supplementation of choline chloride (Sigma‐Aldrich, St. Louis, MO, USA). Supplementation of choline chloride (dissolved in 2 ml of saline, 600 mg/kg/d) was carried out by intravenous infusion for 2 hrs daily, conducted right before TPN infusion as described previously 24. The TPN group also received infusion with the same saline as a vehicle control (the amount used to dissolve choline chloride). The sham group received exactly the same process and were fed with AIN‐93G chow *ad libitum* that contains choline bitartrate (2.5 g/kg diet, 41.1% choline 25). All animals were euthanized at 7 days using CO_2_. The studies were approved by Xin Hua Hospital Animal Use Committee. Composition of TPN solution is presented in Table 1.

**Table 1:** composition of TPN solution

Components	Volume (ml)	kcal	% of Calories
50% glucose	40	80.0	64.44
8.5% amino acids	30	10.2	7.83
20% OOLE (Clinoleic)	20	40.0	30.72
10% sodium chloride	3		
10% potassium chloride	2		
10% calcium gluconate	2		
Water‐soluble vitamins (Soluvit)	0.2		
Fat‐soluble vitamins (Vitalipid)	0.2		
Multi‐trace elements injection	0.2		
Sodium glycerophosphate injection	0.2		
Total	97.8	130.2	100

### Sample collection

The entire small intestine was carefully removed and placed on ice. The lumen of the intestine was flushed with 20 ml of ice‐cold PBS to clear the intestinal lumen. The first 5 cm of jejunum was discarded, and then, 2 cm of jejunum was taken for histology, another 10 cm for the preparation of mucosal content (for RNA extraction and Western blot analysis).

### Tunel assay

Tunel assay was performed using an Apoptosis *In Situ* Detection Kit (Wako, Osaka, Japan) according to the manufacturer's protocol. Briefly, the specimens were deparaffinized and incubated with 20 g/ml proteinase K at 37°C for 20 min., followed by incubation with 100 μl of TdT reaction solution at 37°C for 25 min. Afterwards, the specimens were covered with 100 μl of Alexa Fluor 488‐conjugated antibody solution and counterstained with PI. A minimum of 30 villi were examined, and the number of Tunel‐positive cells was counted. The Tunel‐positive rate was expressed as the ratio of the number of Tunel‐positive cells per villus to the total number of cells.

### Intestinal morphology assessment and immunofluorescence staining

A minimum of 30 villi were examined for the measurement of villus height and crypt depth. Data were analysed with LAS AF LITE image processing software (Leica, Germany). The specimens were stained using standard immunofluorescence procedures. For the detection of CELF1, sections were blocked with 5% BSA for 1 hr at room temperature, followed by incubation with a primary antibody reactive to CELF1 (1:200; Santa Cruz Biotechnology, Santa Cruz, CA, USA) overnight at 4°C. Afterwards, the specimens were incubated with 100 μl of Alexa Fluor 555‐conjugated antibody and counterstained with DAPI. Images were visualized using Leica DMI6000B fluorescence microscopy with LAS AF LITE image processing software (Leica).

### Determination of plasma choline, betaine and phosphocholine concentrations

Plasma‐free choline, betaine and phosphocholine were quantified by HPLC‐MS/MS (Thermo Finnigan, Waltham, MA, USA) as previously described with small modifications 26. Briefly, deuterium‐labelled internal standards of all the analytes (Isotec; Sigma‐Aldrich) were added to 50 μl plasma. Metabolites were separated by using HPLC with an Alltech Solvent Miser Silica analytical column (2.1 × 150 mm, 5 μm; Alltech, Deerfield, IL, USA), and the mass spectrometer was conducted in positive ion electrospray mode. The parention/daughter ion fragments monitored for choline compounds were m/z 104/60 (choline), m/z 184/125/86 (phosphocholine), and m/z 118/59 (betaine). The m/z monitored for deuterium‐labelled internal standards was 113/69 for d9‐choline, 193/125/95 for d9‐phosphocholine and 127/68 for d9‐betaine.

### Cell culture and regents

Caco‐2 cells (American Type Culture Collection, USA) were cultured in Dulbecco's modified Eagle's medium supplemented with 10% foetal bovine serum, at 37°C in a humidified atmosphere containing 5% CO_2_. The medium was changed every 2 days. The cell culture reagents were obtained from Life Technologies (Shanghai, China). Experimental OOLE was derived from a commercial product (Clinoleic, 20% LCT, Baxter Healthcare, China). Choline chloride was purchased from Sigma‐Aldrich (St Louis, MO, USA). Mouse anti CELF1, Bcl‐2, RCK and GAPDH were purchased from Santa Cruz (Shanghai, China). Goat anti Ago2 was also purchased from Santa Cruz. Rabbit anti AIF and Bax were purchased from Proteintech (Shanghai, China). All other chemicals were purchased from Sigma‐Aldrich (Shanghai, China).

### Cell treatment

Caco‐2 cells were exposed to 1% OOLE to induce the up‐regulation of CELF1 and cell apoptosis as described previously 5. Supplementary choline (at 500 μM) was simultaneously added to the medium to investigate its impact on CELF1 expression and apoptosis in OOLE‐treated cells. Moreover, cells were exposed to various concentrations of choline (at 20, 50, 100, 200 and 500 μM) for 6–24 hrs to test the effect of dosage and incubation time on CELF1 expression.

### Apoptosis assessment and determination of mitochondrial membrane potential (MMP)

Apoptosis was determined by flow cytometry using the FITC‐Annexin V apoptosis detection kit according to the manufacturer's protocol. Briefly, cells were stained with FITC‐Annexin V (final concentration, 1 μg/ml) and PI (final concentration, 5 μg/ml), followed by analysis with a FACSCalibur flow cytometer (BD Biosciences, San Jose, CA, USA). A total 1 × 10^4^ cells were analysed per sample. MMP was determined by JC‐1, a fluorescent dye capable of selectively entering mitochondria to form monomers that emit green fluorescence at low MMP and to form JC‐1 aggregates that emit red fluorescence at high MMP. The signal intensity of green/red fluorescence was determined by fluorescence microscopy and fluorescence spectrophotometer (at 490/530 nm for green fluorescence and 525/590 nm for red fluorescence). Disruption or loss of MMP was expressed as the ratio of aggregates (red fluorescence) to monomer (green fluorescence).

### Quantitative real‐time PCR (Q‐PCR)

Total RNA was extracted using Trizol isolation method (Life Technology, Carlsbad, CA, USA). The amplification programme consisted of activation at 95°C for 5 min., followed by 35 amplification cycles, each consisting of 95°C for 15 sec. then 60°C for 1 min. The sequences of primers used in this study were as follows: human CELF1: 5′‐TCCTGCCGTTTGTTCATCGTT‐3′ (forward) and 5′‐ TTTCCCCTTCAGCAGTCGTTC‐3′ (reverse); human GAPDH: 5′‐ TATTGTTGCCATCAATGACCC ‐3′ (forward) and 5′‐ACTCCACGACGTACTCAGC‐3′ (reverse); Rat CELF1: 5′‐TTTTGTTACATTTTACACCCGTA‐3′ (forward) and 5′‐CGAAGAAAACATGACTCGGAT‐3′ (reverse); rat GAPDH: 5′‐CAAAGTGGACATTGTTGCCAT‐3′ (forward) and 5′‐AGACGCCAGTAGACTCCACGA‐3′ (reverse). Quantitative real‐time PCR and data analysis were performed using PIKO96 (Thermo, Germany).

### Western blot analysis

For *in vivo* studies, intestinal epithelial cells were prepared using a modification of Grossmann's technique 27. Briefly, after several washes with ice‐cold PBS, the epithelial cell layer was scraped from the submucosa with a scraper, collected in 1 2 ml tube for homogenate using MagNa Lyser (Roche, Penzberg, Germany). For *in vitro* studies, cells were washed twice with PBS and lysed on ice for 30 min. in 100 μl of RIPA buffer. Protein concentrations were determined using a BCA protein assay kit (Thermo , Rockford, USA). Aliquots of the lysates (30 μg of protein) were loaded, and immunodetection was performed with enhanced chemiluminescence detection system (Thermo, Shanghai, China).

### Immunofluorescence staining

Cells were fixed in 4% formaldehyde at room temperature for 15 min., and permeabilized with 0.3% Triton‐X100 in PBS for 15 min. After blocking, cells were incubated with antibodies against CELF1 and AIF at 4°C overnight, followed by incubation with Alexa Fluor 488 or Alexa Fluor 555‐conjugated secondary antibodies for 1 hr at room temperature. Images were visualized using Leica DMI6000B fluorescence microscopy with LAS AF LITE image processing software (Leica).

### Ribonucleoprotein‐immunoprecipitation (RNP‐IP) assays

For assessment of the association of endogenous RCK or Ago2 with CELF1 mRNA, RNP‐IP complexes were performed as previously described 28. Twenty million cells per sample were collected, and lysates were used for IP at 4°C overnight in the presence of excess (30 μg) IP antibody against RCK or Ago2, or IgG (negative control). RNA in the IP materials was analysed by Q‐PCR to detect the presence of CELF1 mRNAs.

### mRNA stability assay

Caco‐2 cells were treated with Actinomycin D (Act D, at 5 μg/ml) for 0–12 hrs. The amount of mRNA at 0 hr time‐point was set as 100%, and thereafter, the percentage of normalized CELF1 mRNA levels *versus* time was plotted, based on which the half‐time of CELF1 transcripts was calculated.

### Analysis of newly synthesized protein

Nascent synthesis of CELF1 protein was detected by Click‐iT AHA protein analysis detection kit (thermo) according to the manufacturer's protocol. Briefly, cells were incubated in methionine‐free medium and then exposed to L‐azidohomoalanine. After mixing of cell lysates with the reaction buffer containing CuSO_4_ and biotin/alkyne reagent for 20 min., the biotinalkyne/azide‐modified protein complex was pulled down using paramagnetic streptavidin‐conjugated Dynabeads. The pull‐down material was resolved by 10% SDS‐PAGE and analysed by Western blot.

### Statistical analysis

Data are expressed as means ± standard deviation (S.D.) from three to six samples. The significance of the difference between means was determined by Student's *t*‐test; *P* < 0.05 was considered significant.

## Results

### Supplementary choline alleviated TPN‐induced enterocyte apoptosis and down‐regulated the expression of CELF1 *in vivo*

**Figure 1:**
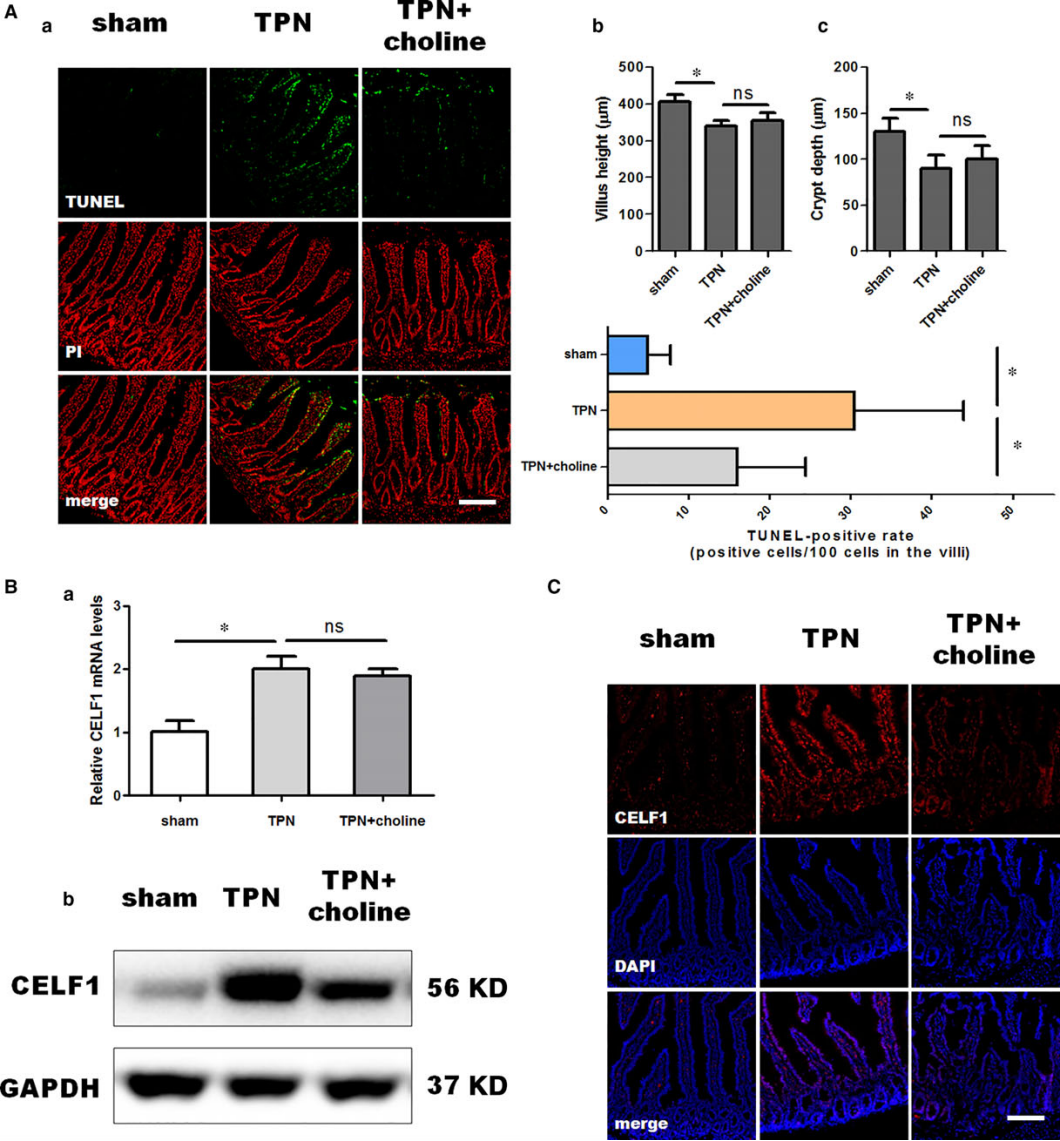
Supplementary choline alleviated TPN‐induced enterocyte apoptosis and down‐regulated the expression of CELF1 *in vivo*. (**A**) Tunel assay (a) and morphologic changes in the villus height (b) and crypt depth (c). Rats were maintained on TPN for 7 days as described in material and methods. Enterocyte apoptosis was determined by Tunel assay. Scale bar = 50 μm. Note that TPN significantly increased the Tunel‐positive rate in comparison with the sham group, which was alleviated by supplementary choline. No obvious differences in the villus height and crypt depth were observed between TPN and TPN with supplementary choline. Data were presented as mean ± S.D. *P < 0.05 (*n* = 5–6/group). (**B**) Changes in the expression of CELF1 responding to supplementary choline in TPN rats. The mRNA levels of CELF1 (a), representative immunoblots of CELF1 (b) and representative immunofluorescence staining of CELF1 (c) in the intestinal mucosa. Notably, supplementary choline did not alter the mRNA expression of CELF1 in comparison to TPN alone, but significantly down‐regulated CELF1 expression on protein levels. Data were presented as mean±S.D. *P < 0.05 (*n* = 5–6/group). Scale bar = 50 μm.

As indicated by Tunel assay, a robust staining of Tunel‐positive cells was observed along the villus of TPN rats, which was obviously alleviated by supplementary choline. The Tunel‐positive rate in the intestinal villus of the sham group, the TPN group and TPN+choline group was 5 ± 2%, 30 ± 10% (*P* < 0.05 compared to sham) and 16 ± 6% (*P* < 0.05 compared to TPN), respectively (Fig. 1A‐a). However, supplementary choline exhibited no significant impacts on villus height (*P* > 0.05 compared to TPN, Fig. 1A‐b) and crypt depth (*P* > 0.05 compared to TPN, Fig. 1A‐c), suggesting that supplementary choline specifically influenced enterocyte apoptosis rather than enterocyte proliferation. Notably, supplementary choline also decreased the expression of CELF1 compared with TPN. Interestingly, however, only the protein expression of CELF1 was obviously suppressed by supplementary choline, while no significant alterations in the mRNA levels of CELF1 were observed in the TPN+choline group compared to TPN alone (Fig. 1B). Collectively, these results indicated a potential role of CELF1 contributing to the protective effect of choline against TPN‐induced enterocyte apoptosis *in vivo*.

### Supplementary choline alleviated OOLE‐induced enterocyte apoptosis *in vitro*

**Figure 2:**
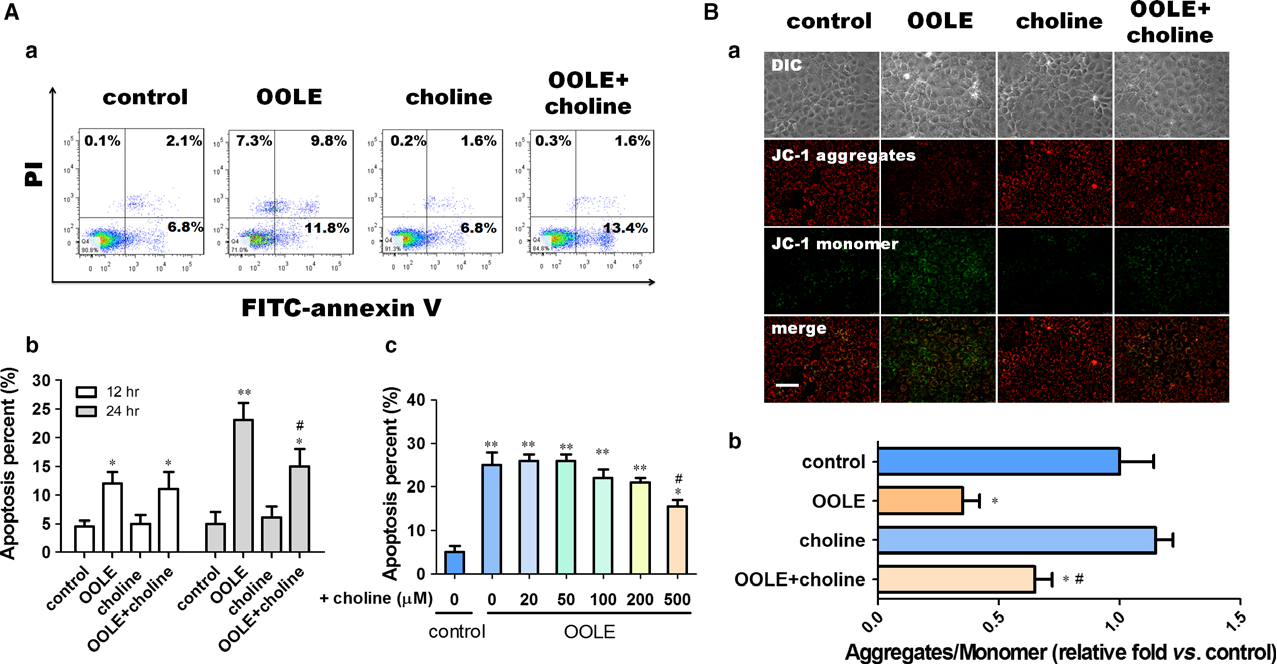
Supplementary choline alleviated OOLE‐induced enterocyte apoptosis *in vitro*. (**A**) Apoptosis determined by Annexin V/PI staining. Caco‐2 cells were treated with OOLE (1%) alone, choline alone (500 μM) and OOLE (1%) with supplementary choline (500 μM), respectively. Apoptosis was determined 24 hrs post‐treatment, and original dot plots are shown (a). The graphs represent typical donors and are representative for three different experiments. Apoptotic percentages in cells treated with supplementary choline (500 μM) for 12–24 hrs were presented within histograms (b). Apoptotic percentages in cells treated with supplementary choline at varying doses for 24 hrs were presented within histograms (c). Values are mean ± S.D. of data from three experiments.*P < 0.05. **P < 0.01 compared to control; ^#^ *P* < 0.05 compared to OOLE alone. (**B**) Mitochondrial membrane potential determined by JC‐1. Caco‐2 cells were treated as described above. Representative images showing JC‐1 monomers (green) and aggregates (red) are presented in (a). Scale bar = 50 μm. The relative ratios (aggregates/monomer) were also determined using a fluorescence spectrophotometer as described in material and methods. Notably, OOLE‐induced disruption of MMP was significantly attenuated by supplementary choline. Values are mean ± S.D. of data from three experiments. *P < 0.05. **P < 0.01 compared to control; ^#^ *P* < 0.05 compared to OOLE alone.

Caco‐2 cells were treated with OOLE (1%) and supplementary choline (500 μM) for 24 hrs. As indicated by Annexin V/PI staining, treatment with OOLE significantly increased the apoptotic rate in comparison with control. Although choline *per se* elicited no significant impact on apoptosis under normal condition, supplementary choline in OOLE substantially alleviated OOLE‐induced apoptosis (Fig. 2A‐a). Interestingly, the anti‐apoptotic effect of supplementary choline was evident only at 24 hrs post‐treatment, although the pro‐apoptotic effect of OOLE was already evident at 12 hrs post‐treatment (Fig. 2A‐b). Moreover, only high‐dose choline (500 μM) elicited the significant anti‐apoptotic effect, while no significant impacts were observed at lower doses (Fig. 2A‐c). Collectively, these results indicated that sufficient accumulation of time and dose is essential for choline to alleviate the apoptosis induced by OOLE *in vitro*. As the intrinsic mitochondria‐initiated pathway is one of the fundamental pathways mediating apoptosis, we further investigated whether changes in the mitochondrial membrane potential (MMP) were changed by supplementary choline. As shown, the intensity of green fluorescence (JC‐monomer) was enhanced, and the intensity of red fluorescence (JC‐aggregates) was reduced in the cells exposed to OOLE. Although choline *per se* elicited no significant impact on MMP under normal condition, supplementary choline in OOLE substantially reduced the intensity of green fluorescence (JC‐monomer) and enhanced the intensity of red fluorescence (JC‐aggregates), compared to OOLE alone. Furthermore, the relative ratios (aggregates/monomer) quantified using a fluorescence spectrophotometer revealed that changes in the MMP were approximately 30% restored by supplementary choline. The relative ratios (aggregates/monomer) of control cells, cells treated with OOLE, cells treated with choline and cells treated with OOLE and supplementary choline were 1.0 ± 0.13, 0.35 ± 0.07 (*P* < 0.05 compared to control), 1.15 ± 0.07 and 0.65 ± 0.08 (*P* < 0.05 compared to OOLE alone), respectively. Taken together, these results indicated that supplementary choline alleviated OOLE‐induced enterocyte apoptosis *via* mitochondria‐initiated pathway.

### Supplementary choline alleviated OOLE‐induced enterocyte apoptosis through suppression of CELF1/AIF pathway *in vitro*

**Figure 3:**
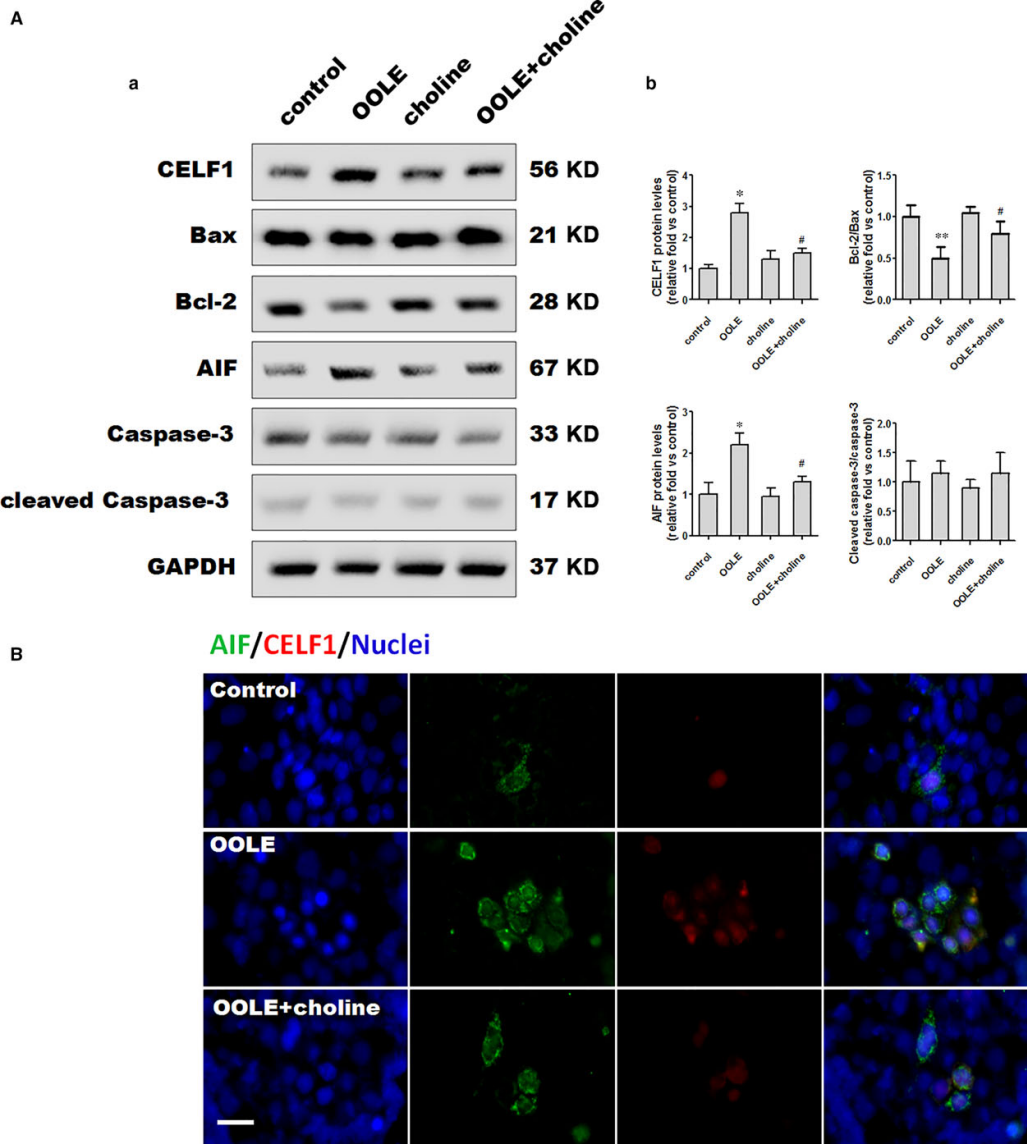
Supplementary choline alleviated OOLE‐induced enterocyte apoptosis through suppression of CELF1/AIF pathway. (**A**) Representative immunoblots of CELF1 and apoptosis‐related proteins. Caco‐2 cells were treated with OOLE (1%) alone, choline alone (500 μM) and OOLE (1%) with supplementary choline (500 μM), respectively. The indicated proteins were analysed 24 hrs after treatment (a), and relative fold was determined by band intensity (b). Notably, supplementary choline significantly suppressed the expression of CELF1, Bcl‐2 and AIF compared to OOLE alone. No obvious alterations were observed in Bax expression and caspase‐3 activation. Values are mean ± S.D. of data from three experiments *P < 0.05. **P < 0.01 compared to control; ^#^ *P* < 0.05 compared to OOLE alone. (**B**) Representative images of CELF1 and AIF staining in Caco‐2 cells. Cells were treated with OOLE (1%) alone and OOLE (1%) with supplementary choline (500 μM) for 24 hrs. Notably, OOLE treatment significantly increased the population of CELF1/AIF double positive cells, which was attenuated by supplementary choline. Scale bar = 75 μm. Three experiments were performed that showed similar results.

Cells were treated with OOLE (1%) and supplementary choline (500 μM) for 24 hrs. As shown, treatment with OOLE led to a significant increase in the expression of CELF1 and AIF, and a significant decrease in the expression of Bcl‐2 in Caco‐2 cells. No significant alterations in the expression of Bax, caspase‐3 and cleaved caspase‐3 were observed. However, changes in the expression of CELF1, Bcl‐2 and AIF were obviously restored by supplementary choline (Fig. 3A). Notably, double‐immunofluorescence staining of CELF1 and AIF clearly indicated that AIF was predominately expressed in cells with high levels of CELF1. Treatment with OOLE significantly increased the population of cells co‐expressing CELF1 and AIF, which was partially restored by supplementary choline (Fig. 3B). Taken together, these results revealed that supplementary choline alleviated OOLE‐induced enterocyte apoptosis *via* suppression of CELF1/AIF pathway.

### Supplementary choline post‐transcriptionally regulated the expression of CELF1

**Figure 4:**
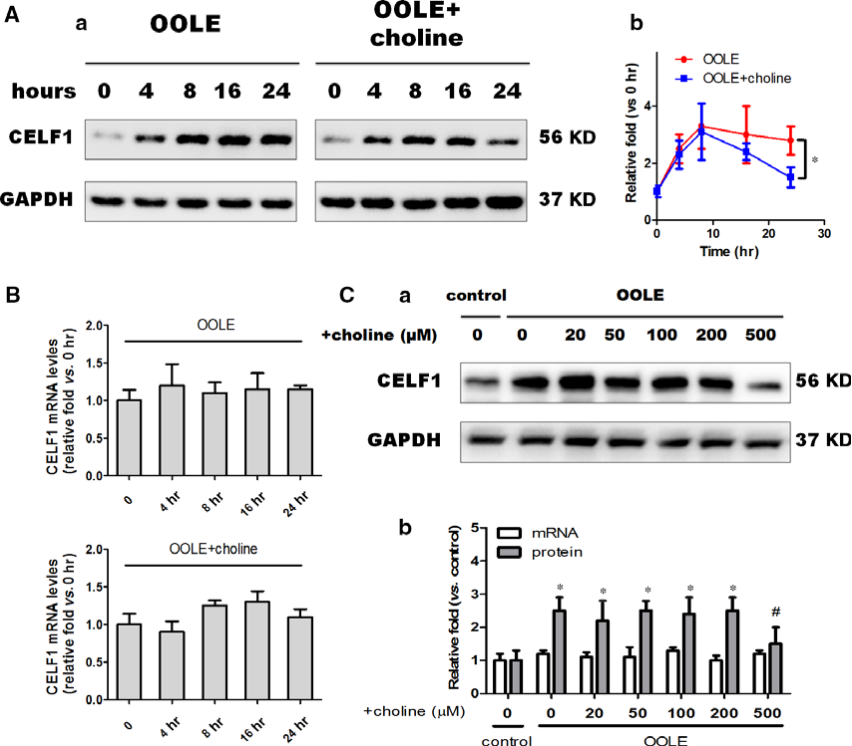
Supplementary choline post‐transcriptionally regulated the expression of CELF1. (**A**) Representative blots of CELF1 in Caco‐2 cells. Cells were treated with OOLE (1%) and supplementary choline (500 μM) for 0, 4, 8, 16 and 24 hrs (a). Changes in the relative fold (determined by band intensity) were plotted along the time axis. Data were normalized to 0 h in each group. Notably, significant differences in CELF1 expression were evident only at 24 hrs post‐treatment. Values are mean ± S.D. of data from three experiments.*P < 0.05. (**B**) CELF1 mRNA levels in the cells described above. Note that the mRNA expression of CELF1 was unchanged by either OOLE alone or OOLE with supplementary choline. Values are mean ± S.D. of data from three experiments. (**C**) Representative blots of CELF1 in cells treated with OOLE (1%) and varying doses of supplementary choline. The protein levels of CELF1 were determined at 24 hrs after treatment (a). Changes in the protein levels (determined by band intensity) and mRNA levels (determined by Q‐PCR) were also presented within histograms (b). Values are mean ± S.D. of data from three experiments. *P < 0.05. compared to control; ^#^ *P* < 0.05 compared to OOLE alone.

As shown, the protein levels of CELF1 were markedly increased by OOLE treatment on a time‐dependent manner. Although no significant differences in the protein expression of CELF1 were observed before 16 hrs post‐treatment, significant suppression of CELF1 expression was observed at 24 hrs post‐treatment by supplementary choline (*P* < 0.05 compared to OOLE alone, Fig. 4A). However, neither treatment with OOLE alone nor treatment with OOLE with supplementary choline altered the mRNA levels of CELF1 (Fig. 4B). Notably, consistent with the results of Figure 2A‐c, only high‐dose choline (500 μM) elicited the significant effect on CELF1 expression, while no significant impacts were observed at lower doses (Fig. 4C). Collectively, these results suggested that (i) sufficient accumulation of time and dose is essential for choline to repress the expression of CELF1; (ii) regulation of CELF1 by supplementary choline may occur at the post‐transcriptional level.

### Supplementary choline induced CELF1 mRNA decay by recruiting CELF1 transcripts to processing bodies

**Figure 5:**
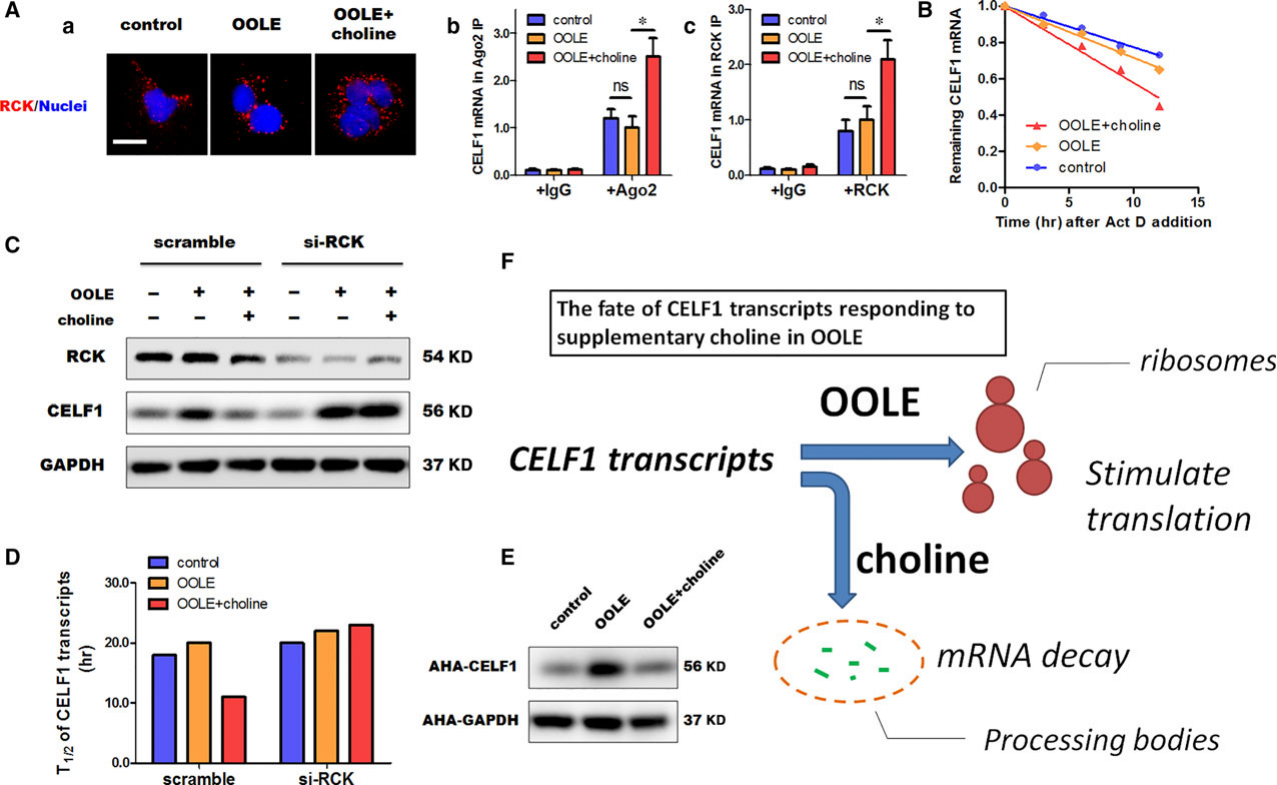
Supplementary choline in OOLE induced CELF1 mRNA decay by recruiting CELF1 transcripts to processing bodies. (**A**) Supplementary choline induced recruitment of CELF1 transcripts to processing bodies. Representative images of RCK staining in Caco‐2 cells (a). RCK was labelled as one of the components of processing bodies. No significant effects on the expression and localization of RCK were observed by OOLE (1%) and choline (500 μM). Scale bar = 75 μm. Three experiments were performed three times that showed similar results. The results of RNP‐IP assays (b, c) revealed that supplementary choline significantly increased the associations of CELF1 transcripts with Ago2 and RCK in compared with OOLE alone. Data were normalized to OOLE alone, and shown as mean ± S.D. (*n* = 3). *P < 0.05. (**B**) Supplementary choline induced CELF1 mRNA decay. Caco‐2 cells were exposed to OOLE (1%) and choline (500 μM) for 12 hrs and then further treated with the RNA polymerase inhibitor actinomycin D for the indicated periods (0, 3, 6, 9 and 12 hrs). CELF1 mRNA levels were analysed by Q‐PCR. (**C**) Supplementary choline repressed CELF1 translation through RCK. Caco‐2 cells were transfected with si‐RCK, followed by incubation with OOLE (1%) and supplementary choline (500 μM) for 24 hrs. As shown, the effect of choline on CELF1 was abolished by depletion of RCK. Three experiments were performed that showed similar results. (**D**) Half‐time of CELF1 transcripts. Data were presented as mean from triplets in one representative experiment. (**E**) Nascent protein synthesis. Newly translated CELF1 protein was determined using CLICK‐iT protein synthesis kit as described in material and methods. As shown, enhanced nascent protein synthesis of CELF1 was obviously abolished by supplementary choline. (**F**) Schematic representation: OOLE induces CELF1 expression by stimulating translation in ribosomes, while supplementary choline induces CELF1 mRNA decay by recruiting CELF1 transcripts to processing bodies.

RCK and Ago2 are critical resident proteins in processing bodies that accumulate a fraction of translationally silent mRNAs for storage, reversible repression and decay. As indicated in Figure 5A, no significant alterations in the expression of RCK were observed by OOLE or OOLE with supplementary choline, suggesting that the number of processing bodies was not affected by OOLE or choline. However, it was found that supplementary choline enhanced the association of CELF1 mRNA with processing bodies, as indicated by an elevation in the levels of CELF1 mRNA in RCK as well as Ago2 compared to OOLE alone (Fig. 5A). As a result, the CELF1 mRNA stability was decreased by supplementary choline compared to OOLE alone, as indicated by the remaining CELF1 mRNA levels following Act D treatment (Fig. 5B). Moreover, silencing RCK by specific siRNA totally abolished the effects of supplementary choline on the expression of CELF1 (Fig. 5C), as well as on the stability of CELF1 transcripts (shown as the half‐time of CELF1 transcripts, Fig. 5D). Furthermore, nascent protein synthesis using AHA labelling revealed that OOLE‐induced elevation in newly translated CELF1 protein was obviously abolished by supplementary choline (Fig. 5E). Taken together, these results suggested that supplementary choline in OOLE repressed the protein translation of CELF1 and induced CELF1 mRNA decay by recruiting CELF1 transcripts to processing bodies (schematic representation shown in Fig. 5F).

## Discussion

A substantial body of literature implicated that TPN may lead to intestinal atrophy, contributing to the development of TPN‐associated complications such as gut‐derived infections, loss of intestinal immune reactivity and even parenteral nutrition‐associated liver disease (PNALD) 29, 30. Recently, a rodent study revealed that intravenous LEs *per se* might elicit significant effects on intestinal physiology, but little is known regarding the molecular mechanism by which LEs induce the enterocyte apoptosis and how to optimize the TPN strategies accordingly 4. In fact, varying LEs with various compositions of fatty acids may induce apoptosis *via* distinct pathways. In our previous study, we found that OOLE specifically induces apoptosis *via* CELF1/AIF pathway, and thus, this present study was designed to address whether OOLE‐induced enterocyte apoptosis could be alleviated by suppressing CELF1/AIF pathway.

Although choline has been declared as an essential nutrient by the US Institute of Medicine's Food and Nutrition Board since 1998, it is currently not a regular nutrient added in the standard PN formula 31. In fact, intravenous LE only contains a minimal amount of free choline and a small amount of phosphocholine which cannot be converted to choline to any significant degree 32. Importantly, however, it is decreased plasma‐free choline concentration that has been associated with the development of TPN‐associated complications. In our study, although TPN reduced plasma levels of free choline, betaine and phosphocholine, they were successfully restored by supplementary choline (Fig. S1), suggesting that the following changes *in vivo* could be attributed to the recovery of plasma choline concentration under our experimental conditions. CELF1 is a multifunctional RNA‐binding protein contributing to the regulation of many post‐transcriptional processes including alternative splicing, translation, de‐adenylation and mRNA degradation 33. The pro‐apoptotic role of CELF1 in enterocytes, as well as its role in mediating OOLE‐induced apoptosis, has been confirmed in our previous work *in vitro* 5. Additionally, Liu *et al*. also reported that overexpression of CELF1 may contribute to the development of fasting‐induced intestinal atrophy *in vivo* 7. Collectively, these findings suggested a potential role of CELF1 in the pathogenesis of TPN‐induced intestinal atrophy. In the present study, by examining the expression of CELF1 and morphological changes in the intestinal tissues of TPN rats infused with intravenous OOLE and supplementary choline, we revealed a potential association between CELF1 and the protective effect of choline against enterocyte apoptosis *in vivo*. Although surprising, no significant changes were observed after choline supplementation in villus height and crypth depth that morphologically reflect the proliferation of enterocytes, whereas the apoptosis of epithelial cells along the villus was obviously ameliorated by supplementary choline (Fig. 1A). These results suggested that supplementary choline during TPN administration are substantially beneficial to alleviating TPN‐induced intestinal atrophy *in vivo*, in the terms of enterocyte apoptosis rather than enterocyte proliferation. However, one limitation of this study is the undetermined optimal concentration for supplementary choline. As known, choline is an endogenous water‐soluble vitamin, and excessive choline could be easily excreted in urine. Because of the metabolic pool of endogenous choline and its derivatives, it is difficult to achieve a dosage‐dependent attenuation of choline deficiency by performing a dosage‐dependent administration of supplementary choline. Besides, to our knowledge, the definition of ‘mild’, ‘moderate’ or ‘severe’ choline deficiency during TPN support is unavailable currently. Due to the lack of clear definition in clinical and the technical limit on the control of endogenous choline levels, we can only provide experimental evidence to demonstrate the effectiveness of choline against TPN‐associated intestinal atrophy *in vivo*, but we are unable to determine the optimal concentration for supplementary choline currently.

One aspect requires comment here. The dosage of OOLE to induce apoptosis in Caco‐2 cells was set at 1% because it elicited the most significant pro‐apoptotic effect as described in our previous work 5. Supplementation of choline in 1% OOLE was set at 500 μM *in vitro*, because this dosage was approximately equal to supplementation of choline at 600 mg/kg/d in OOLE (60 ml/kg/d) *in vivo*. Our results demonstrated that supplementary choline in OOLE substantially alleviated OOLE‐induced apoptosis in Caco‐2 cells. Notably, the population of late apoptosis was obviously reduced by supplementary choline, suggesting that supplementary choline might alleviate OOLE‐induced apoptosis by postponing the apoptosis process into late phase (Fig. 2A). Furthermore, our results indicated that supplementary choline alleviated OOLE‐induced enterocyte apoptosis *via* mitochondria‐initiated pathway, as disruption of MMP induced by OOLE was obviously ameliorated by supplementary choline (Fig. 2B). Interestingly, our results regarding the effect of supplementary choline on MMP was consistent with that previously described by Guo *et al*. 34, in which they found that the disruption of MMP is an upstream event in choline deficiency‐induced apoptosis, and mitochondrial dysfunction plays a key role in mediating choline deficiency‐induced apoptosis in CWSV‐1 cells. Taken together, these results consolidated the effect of supplementary choline on the modulation of apoptosis and mitochondrial dysfunction *in vitro*.

Mechanisms that account for the attenuation effect of choline on OOLE‐induced mitochondrial dysfunction remain unclear currently. As known, excessive cellular lipid accumulation can enhance the susceptibility to lipid peroxidation and ROS (reactive oxygen species) production 35, leading to oxidative stress and mitochondrial dysfunction with ATP reduction and decreased lipid catabolism 36. On the other hand, some studies that declared choline as an anti‐ROS factor through relieving activation of pro‐inflammatory responses 37, 38 suggested the possibility that choline may attenuate OOLE‐induced mitochondrial injury by reducing ROS generation. However, the results that supplementary choline failed to attenuate the apoptosis induced by either SOLE or FOLE in our preliminary studies (data not shown) suggested that supplementary choline may specifically attenuate OOLE‐induced apoptosis *via* an OOLE‐related ‘specific’ pathway instead of ‘unspecific’ lipid peroxidation and ROS generation. Given that OOLE‐induced apoptosis is partially mediated *via* CELF1/AIF pathway, we examined whether the expression of CELF1 and AIF was changed when supplementary choline was added. Notably, up‐regulation of CELF1 and AIF was attenuated by approximately 50% and approximately 54% when choline was supplemented (Fig. 3A). Consistently, the representative images of immunofluorscence clearly revealed a strong association between CELF1 and AIF in the cells exposed to OOLE, which visually confirmed the involvement of CELF1/AIF pathway in OOLE‐induced apoptosis (Fig. 3B). Additionally, we also found that decreased expression of Bcl‐2 was obviously attenuated by choline. As dynamic balance of Bax and Bcl‐2 modulates the release of AIF from mitochondria to induce the apoptotic process in the nucleus, this result suggested that choline may not only suppress AIF expression through down‐regulation of CELF1, but also inhibit the nuclear translocation of AIF through up‐regulation of Bcl‐2.

One important issue of this study was that sufficient accumulation of time and dose is essential for choline to repress the expression of CELF1. As indicated in Figure 4, only treatment with high dose of choline (500 μM) exhibited significant repression effect on CELF1 expression. On the other hand, treatment for 24 hrs was necessary for choline to repress CELF1 expression, whereas the expression of CELF1 could be induced by OOLE within 4 hrs. Obviously, under our experimental condition, in combination with the results mentioned above, these findings suggested that the anti‐apoptotic effect of supplementary choline comes much later than the pro‐apoptotic effect of OOLE. Therefore, it seems better to consider the supplementation of choline as soon as possible for patients receiving TPN support with low plasma free choline concentration.

Finally, we examined the molecular mechanism underlying the regulation of CELF1 by supplementary choline. Our results indicated that a post‐transcriptional mechanism may be involved in the regulation of CELF1 expression. Processing bodies are distinct cytoplasmic granules that accumulate a fraction of translationally silent mRNAs and are defined as the sites of mRNA storage, reversible mRNA repression and mRNA decay 39, 40, 41. Our previous study revealed that OOLE‐induced up‐regulation of CELF1 is attributed to direct stimulation on the protein translation of CELF1 in ribosomes. Interestingly, the results in this study indicated that supplementary choline may repress the protein translation of CELF1 by recruiting CELF1 mRNA to processing bodies (Fig. 5). However, a key question that remains to be addressed is how it affects the recruitment CELF1 mRNA to processing bodies. Indeed, our data may be not convincing enough to fully clarify the underlying mechanism currently, but we suppose that changes in the microRNA pool by choline may play an important part. As known, choline is an essential nutrient that plays an important role in lipid metabolism and DNA methylation, and a number of studies have recently demonstrated that the expression of microRNAs could be regulated by supplementary choline or choline deficiency 42, 43. Specific microRNAs binding to the CELF1 mRNA may be activated by choline, leading to mciroRNA‐mediated gene silencing in processing bodies. Although the potential candidate is currently unknown, our data revealed the possibility that competition between OOLE and choline may contribute to the determination of CELF1 mRNA fate: translated in ribosomes or degraded in processing bodies.

Nevertheless, one of the limitations in this study is that the pro‐apoptotic role of CELF1 has not been confirmed *in vivo*, and thus, it is still unclear to what extent changes in CELF1 expression contribute to the protective effect of choline under TPN support. Therefore, it would be of great significance to further consolidate the role of CELF1 in future studies using CELF1‐knockout animals.

In conclusion, supplementary choline could alleviate OOLE‐induced enterocyte apoptosis both *in vivo* and *in vitro*. Supplementary choline may repress the translation of CELF1 by recruiting CELF1 mRNA to processing bodies, thereby suppressing CELF1/AIF pathway. Our findings provide experimental evidence that the addition of choline to clinical TPN regiments may have therapeutic potential for protecting against TPN‐induced intestinal atrophy.

## Conflict of interest

The authors have declared no conflict of interest.

## Supporting information


**Figure S1** Plasma concentration of choline, betaine and phosphocholine in TPN rats.
