# Supplementary figures and images for: Supplementary choline attenuates olive oil lipid emulsion‐induced enterocyte apoptosis through suppression of CELF1/AIF pathway
Source: J Cell Mol Med. 2017 Nov 6;22(3):1562–73. doi: 10.1111/jcmm.13430 (PMC5824412; doi:10.1111/jcmm.13430)

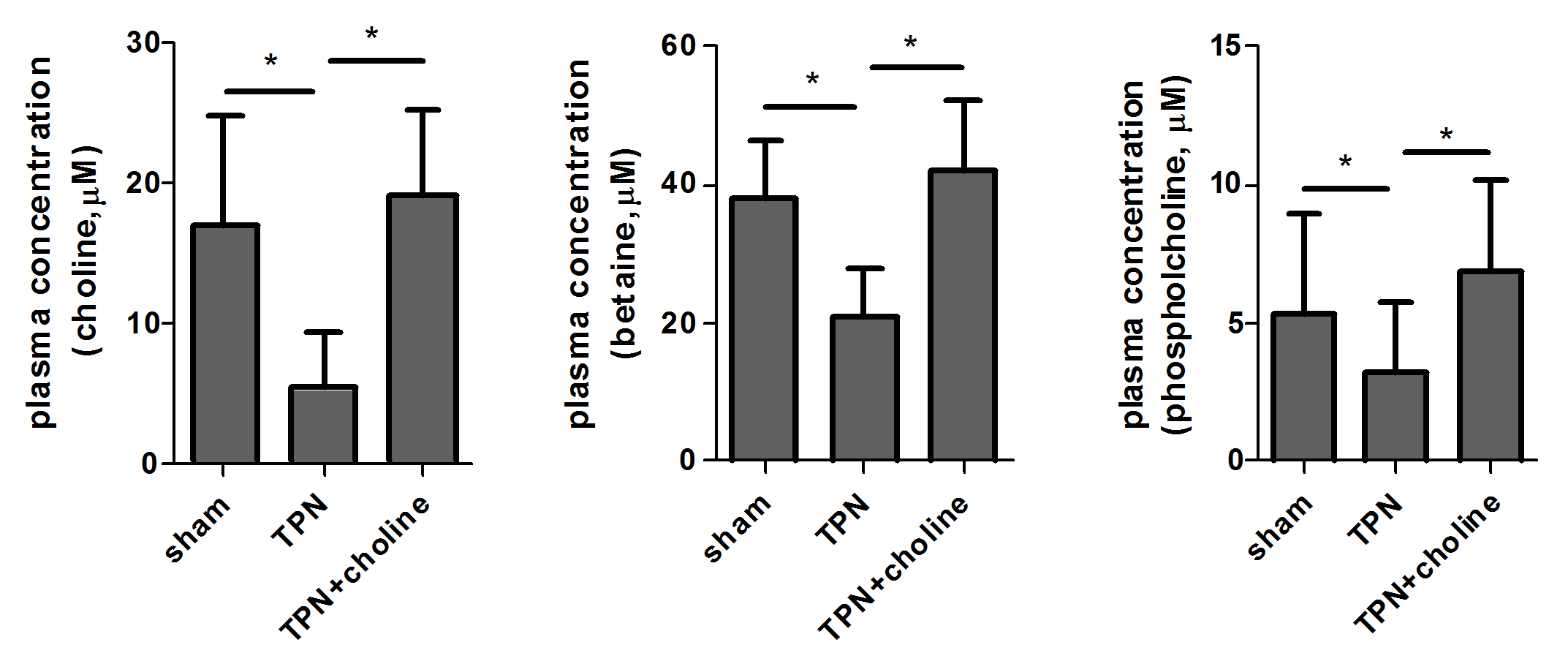

Supplement: Supplementary file 1 — Figure S1 Plasma concentration of choline, betaine and phosphocholine in TPN rats. [file JCMM-22-1562-s001.tif]
